# Machine learning polysomnographically-derived electroencephalography biomarkers predictive of epworth sleepiness scale

**DOI:** 10.1038/s41598-023-34716-5

**Published:** 2023-06-05

**Authors:** Matheus Araujo, Samer Ghosn, Lu Wang, Nengah Hariadi, Samantha Wells, Carl Y. Saab, Reena Mehra

**Affiliations:** 1 Sleep Disorders Center, Neurological Institute, Cleveland Clinic Foundation, Cleveland, OH USA; 2 Department of Biomedical Engineering, Cleveland Clinic Foundation, Cleveland, OH USA; 3 Quantitative Health Sciences, Lerner Research Institute, Cleveland Clinic Foundation, Cleveland, OH USA; 4 Metro Health Medical Center, Cleveland, OH USA; 5 Department of Biomedical Engineering, Brown University, Providence, RI USA; 6 Respiratory Institute, Cleveland Clinic Foundation, Cleveland, OH USA; 7 Cardiovascular and Metabolic Sciences, Lerner Research Institute, Cleveland Clinic Foundation, Cleveland, OH USA; 8 Heart and Vascular Institute, Cleveland Clinic Foundation, Cleveland, OH USA

**Keywords:** Sleep disorders, Predictive markers

## Abstract

Excessive daytime sleepiness (EDS) causes difficulty in concentrating and continuous fatigue during the day. In the clinical setting, the assessment and diagnosis of EDS rely mostly on subjective questionnaires and verbal reports, which compromises the reliability of clinical diagnosis and the ability to robustly discern candidacy for available therapies and track treatment response. In this study, we used a computational pipeline for the automated, rapid, high-throughput, and objective analysis of previously collected encephalography (EEG) data to identify surrogate biomarkers for EDS, thereby defining the quantitative EEG changes in individuals with high Epworth Sleepiness Scale (ESS) (n = 31), compared to a group of individuals with low ESS (n = 41) at the Cleveland Clinic. The epochs of EEG analyzed were extracted from a large overnight polysomnogram registry during the most proximate period of wakefulness. Signal processing of EEG showed significantly different EEG features in the low ESS group compared to high ESS, including enhanced power in the alpha and beta bands and attenuation in the delta and theta bands. Our machine learning (ML) algorithms trained on the binary classification of high vs. low ESS reached an accuracy of 80.2%, precision of 79.2%, recall of 73.8% and specificity of 85.3%. Moreover, we ruled out the effects of confounding clinical variables by evaluating the statistical contribution of these variables on our ML models. These results indicate that EEG data contain information in the form of rhythmic activity that could be leveraged for the quantitative assessment of EDS using ML.

## Introduction

Excessive daytime sleepiness (EDS) occurs when staying awake or alert is a progressive challenge for the individual. This condition is not only inherent to hypersomnia disorders such as narcolepsy, idiopathic hypersomnia and sleep disordered breathing, but also can be associated with a range of clinical factors, including metabolic and neurological diseases, ultimately translating to impairment of voluntary activities during the day or night^1^. EDS has become a significant public concern when associated with fatigue, costing more than $135 billion annually in health-related lost productivity in the United States^2^. In addition to the financial cost, the individual perception of the difficulty to concentrate and the deterioration of the brain response to audio, visual, and other stimulation motivates the search for a non-invasive biomarker that can help identify EDS to provide effective treatment. Seeking to find associations between sleepiness and its intertwined dynamics in the central nervous system (CNS), we tested the hypothesis that EEG data contain information in the form of rhythmic activity that could be leveraged for the quantitative assessment of EDS using machine learning (ML).

Day-time sleepiness affects the CNS and leads to changes in brain function and rhythms. Previous studies reported desynchronization between the left and right hemispheres under mental fatigue^3^, and imaging data suggest altered functional connectivity between thalamus and cortex^4^. In fact, EEG has shown promising results in identifying EDS biomarkers, especially for fatigue versus alert state classification during activities such as driving using portable EEG devices^5^ and for predicting driving reaction time^6^. In the clinic, subjective sleepiness symptoms can be assessed by the Epworth Sleepiness Scale (ESS), which is the state-of-practice self-report to quantify EDS or dozing propensity, and in general, it is highly correlated to the standard of care sleepiness measurements such as the multiple sleep latency test^7^. However, current diagnostic methods remain essentially subjective, because they rely on questionnaires and verbal reports.

In this study, we recorded resting state EEG from awake human subjects obtained from the Cleveland Clinic registry of overnight polysomnograms and pre-processed the data using an automated artifact detection algorithm that our team has previously developed^8^. Following a statistically-guided approach for EEG feature selection, we trained a ML algorithm to perform a binary classification of low versus high EDS. We further complement our study with a statistical analysis of the contribution of confounding clinical variables to our ML binary classifier.

## Methods

### Study population

We leveraged the Cleveland Clinic Sleep Registry, a collection of multimodal physiologic data, including continuous overnight EEG housing sleep studies. Data from this biophysiological repository was extracted for the purposes of this study with a focus on overnight polysomnograms or split night sleep studies. We abstracted polysomnogram data from a merged initiative of Cleveland Clinic’s distributed sleep centers to capture those with severe hypersomnolence and those without symptoms of EDS. To serve as the analytic sample for the work, we identified from a total of 72 patients, 31 patients that had severe EDS, defined by an ESS greater or equal to 20 and 41 patients that had no EDS, defined by an ESS less than 5. This study (#22–135) was approved by Cleveland Clinic Institutional Review Board (IRB) Federal Wide Assurance (FWA 00005367) on 2/17/2022 as Exempt Human Subject Research, which granted a waiver of Informed Consent.

### Awake data acquisition

The polysomnogram studies were conducted in accordance with the American Academy of Sleep Medicine (AASM) guidelines using Polysmith software version 10 (Nihon Kohden). Signals were recorded using a standard 10–20 EEG montage. Only 6 referenced channels were selected for analysis, in some subjects, individual channels are referenced by ‘M1’ or ‘M2’. Odd channel numbers (located on the left side of the brain) are referenced by M1 and even channel numbers (located on the right side of the brain) are referenced by M2 in an ideal setting, rarely on cases of reference malfunction, the other side reference was used. The channels used are ‘F3’, ‘F4’, ‘C3’, ‘C4’, ‘O1’, and ‘O2’. The EEG was scanned and only the cleanest signal of 3 continuous minutes interval between “lights out” and the first stage of sleep “N1” was selected, this is the period we define as “awake” epochs in the context for this study. The cleanest signal is based on the number of artifacts. “Lights out” is a log event added by the technician and it means that troubleshooting and the bio calibration has been done, and that the sleep study is ready to be recorded. The awake epochs align with AASM guidelines, which requires that more than 50% of the epoch consists of alpha frequency activity.

### EEG preprocessing

Pre-processing of EEG data, feature extraction, statistics, and ML were performed using MATLAB (MathWorks). Since sleepiness is a feature of wakefulness, only EEGs during resting state wakefulness were analyzed, defined as the EEG recording time when subjects were awake prior to the sleep study.

The “awake” epochs selected were scored and chosen as wake manually and annotated electronically in the EEG file. Sleep studies conducted in the Cleveland Clinic Sleep Disorders Laboratory were manually scored for sleep staging including sleep and wake using American Academy of Sleep Medicine scoring rules. After this step, the algorithm developed was based upon EEG data extracted automatically from the wake epochs of the sleep studies. In this respect, we considered the EEG analysis as being automated. Greater than 50% of the epoch was comprised of alpha frequency activity without microsleeps. EEG was collected at a sampling rate of 200 Hz. A high-pass filter with a passband frequency of 1 Hz and a notch filter with a stop-band of 57.5–62.5 Hz were applied to all recordings. All EEG recordings were first visually inspected to confirm overall signal quality for each channel; channels considered to be of low or irretrievable quality were excluded from the study. Waveforms in each channel were further divided into 1-s epochs, and each epoch was tested for the presence of artifacts using a previously validated support vector machine (SVM)^8^. Epochs containing artifacts were excluded.

### Statistical analyses

From the remaining artifact-free epochs of each recording, the following features were extracted: band-wise PSD for all channels and band-wise Phase-Amplitude Coupling (PAC). To create the band-wise PSD, a periodogram was gathered from artifact-free epochs for each channel, and then these periodograms were averaged together for each channel within each subject. These averaged periodograms were normalized by dividing each frequency bin by the sum of all bins from 3 to 30 Hz. The normalized PSD was used to calculate the band-wise PSD by taking the average of all bins within each of the following four frequency bands: Delta (1–4 Hz), Theta (5–9 Hz), Alpha (10–13 Hz), Beta (14–32 Hz), and low Gamma (33–52 Hz). This yielded 5 PSD features for every channel included.

The Band by Channel PSD, is similar to the band-Wise PSD. The only difference is that channels are not averaged; each channel is considered separately. This yielded a total of 30 features (5 bands × 6 channels) to be considered.

PAC was calculated using the Modulation Index (MI) method^9^. The center frequencies used for the phase included all the even numbers from 2 to 20. The center frequencies used for amplitude included all multiples of 3 from 30 to 54. MI was measured for each pair of phase and amplitude frequencies (90 total pairs) for each channel, including only those time points for which there were 5 or more consecutive artifact-free eyes-open epochs. This yielded a 9 × 10 MI matrix, for every channel of every subject, with each row corresponding to one phase center frequency and each column corresponding to one amplitude center frequency. This MI matrix was converted to band-wise PAC for the following 4 pairs of bands: Delta—low gamma, Theta—low Gamma, Alpha—low Gamma, and Beta—low Gamma. Other band-wise PAC were computed for the following 4 pairs of bands: Delta—Medium Gamma, Theta—Medium Gamma, Alpha—Medium Gamma, and Beta—Medium Gamma. This conversion was accomplished by averaging across the appropriate regions of the MI matrix. This yielded 4 PAC features for each channel and a maximum of 24 PAC features per subject (4 band-pairs × 6 channels).

Bandwise coherence was calculated from each of 15 unique channel pairs by averaging the coherence (MATLAB function *mscohere*) from each artifact-free epoch during the recordings for both channels in a given pair. This average coherence was divided into five bands in the same manner as PSDs. This yielded 5 coherence features for each channel pair, and a maximum of 75 coherence features per subject (5 bands × 15 channel pairs), coherence values were not computed from channel pairs for which one or both of the channels included artifacts.

We used paired two-tailed *t* tests to compare the band-wise Power Spectral Density (PSDs) between the high ESS and low ESS groups^10^. We used two-tailed Wilcoxon rank-sum tests to compare the band-wise phase-amplitude coupling (PAC) from the two groups for each of 4 band pairs and 6 channels. We chose a non-parametric statistical test for PAC because values are constrained between 0 and 1 and are therefore less likely to follow a normal distribution, as required by Student's *t* test. Statistical significance was established throughout at *p* value < 0.05. As individual testing was conducted, and the purpose of the statistical testing was primarily the selection of features for subsequent ML (rather than testing of a null hypothesis), there was no adjustment for multiple comparisons^11,12^. Unlike simultaneous family testing with a joint null hypothesis comprising two or more null hypotheses, individual testing is utilized to make a decision about one null hypothesis. As each test provides only one opportunity to make a Type I error, the alpha level does not require lowering.

Although it is common in the literature to include confounding factors as input in a regression model for further adjustment, this approach is inadequate in ML classifiers that can learn complicated non-linear relationships between the input and output. Therefore, we performed the post-hoc analysis described in^13^, which proposes controlling for confounds by using traditional regression to compare the extent to which a trained ML prediction itself can explain the target variable, in contrast to the independent performance of confounding variables.

## Results

Our study population was overall middle-aged (mean age 54 years) with a relatively even distribution of men and women, race-based diversity (34.7% African American) and a mild degree of sleep-disordered breathing (apnea–hypopnea index = 13.4). Those with a higher degree of EDS were more likely to be slightly younger, female and more obese with longer total sleep time and a lower percentage of N1 sleep stage. Of note, as reflective of our pre-specified design, those with a high degree of EDS had a mean ESS of 21 ± 1, in contrast, those without EDS had a mean ESS of 2 ± 1. Detailed demographic information, sleep characteristics, and medical history for overall patients and their subsequent division into high ESS and low ESS groups are shown in Table 1.

**Table 1:** Patient Characteristics.

	Overall (N = 72)		Low ESS (N = 41)		High ESS (N = 31)		*p* value
	N		N		N		
Age (yrs)	72	54.4 ± 14.5	41	55.7 ± 14.5	31	52.7 ± 14.5	0.39^a1^
Gender, female	72	35 (48.6)	41	16 (39.0)	31	19 (61.3)	0.061^c^
Ethnicity, Hispanic	72	9 (12.5)	41	5 (12.2)	31	4 (12.9)	0.99^d^
Race	72		41		31		0.097^c^
White		37 (51.4)		25 (61.0)		12 (38.7)	
Black or African American		25 (34.7)		10 (24.4)		15 (48.4)	
Other		10 (13.9)		6 (14.6)		4 (12.9)	
Sleep procedure type	72		41		31		0.92^c^
Polysomnograms		19 (26.4)		11 (26.8)		8 (25.8)	
Split		53 (73.6)		30 (73.2)		23 (74.2)	
Body mass index (BMI), kg/m^2^	72	37.8 ± 9.6	41	36.5 ± 9.4	31	39.5 ± 9.9	0.19^a1^
Epworth sleepiness scale	72	2.0 [1.00, 21.0]	41	2.0 [1.00, 2.0]	31	21.0 [20.0, 22.0]	** < *0.001***^***b***^
Total sleep time, min	72	319.5 [278.0, 371.0]	41	311.0 [242.0, 359.0]	31	330.0 [304.0, 385.0]	***0.031***^***b***^
Apnea Hypopnea index (AHI)	72	13.4 [6.0, 38.7]	41	13.1 [6.4, 36.6]	31	13.4 [5.9, 42.1]	0.66^b^
Obstructive sleep apnea (AHI ≥ 5)	72	61 (84.7)	41	34 (82.9)	31	27 (87.1)	0.75^d^
Hypopnea rule (3% vs. 4%) − 3%	72	43 (59.7)	41	24 (58.5)	31	19 (61.3)	0.81
% Sleep time with SaO_2_ < 90%	72	1.6 [0.15, 10.6]	41	2.0 [0.30, 8.0]	31	1.00 [0.10, 19.7]	0.90^b^
Arousal index	71	22.5 [13.6, 39.9]	40	24.9 [15.6, 39.1]	31	19.9 [11.7, 39.9]	0.44^b^
Mean oxygen saturation	72	93.0 [92.0, 95.0]	41	93.0 [92.0, 94.0]	31	93.0 [91.0, 95.0]	0.58^b^
Minimum oxygen saturation	72	85.5 [75.0, 89.0]	41	86.0 [74.0, 89.0]	31	85.0 [77.0, 89.0]	0.72^b^
Sleep stage % N1	69	6.1 [3.5, 11.0]	39	6.8 [3.9, 13.4]	30	4.5 [2.6, 7.0]	***0.023***^***b***^
Sleep stage % N2	69	67.0 [55.3, 74.7]	39	67.2 [55.2, 74.7]	30	66.7 [55.3, 74.7]	0.96^b^
Sleep stage % N3	68	5.3 [0.00, 15.1]	39	2.4 [0.00, 16.3]	29	8.4 [0.00, 13.9]	0.51^b^
Sleep stage % REM	70	17.9 [12.6, 23.4]	40	17.4 [12.5, 20.1]	30	20.1 [13.1, 26.3]	0.17^b^
Coronary artery disease	72	8 (11.1)	41	4 (9.8)	31	4 (12.9)	0.72^d^
Hypertension	72	44 (61.1)	41	28 (68.3)	31	16 (51.6)	0.15^c^
Heart failure	72	10 (13.9)	41	6 (14.6)	31	4 (12.9)	0.99^d^
Asthma	72	22 (30.6)	41	11 (26.8)	31	11 (35.5)	0.43^c^
COPD/emphysema	72	13 (18.1)	41	4 (9.8)	31	9 (29.0)	***0.035***^***c***^
Cancer	72	11 (15.3)	41	7 (17.1)	31	4 (12.9)	0.75^d^
Diabetes	72	26 (36.1)	41	14 (34.1)	31	12 (38.7)	0.69^c^
Smoking	72		41		31		0.38^d^
No		46 (63.9)		23 (56.1)		23 (74.2)	
Current smoker		7 (9.7)		5 (12.2)		2 (6.5)	
Former smoker		19 (26.4)		13 (31.7)		6 (19.4)	
Smoking pack years	72		41		31		0.092^b^
0.Never smoked		59 (81.9)		31 (75.6)		28 (90.3)	
0–10		4 (5.6)		2 (4.9)		2 (6.5)	
10–30		8 (11.1)		7 (17.1)		1 (3.2)	
30 +		1 (1.4)		1 (2.4)		0 (0.00)	

*p values*: *a*1 *t* test, *a*2 Satterthwaite *t* test, *b* Wilcoxon rank sum test, *c* Pearson's chi-square test, *d* Fisher's exact test.

Significant values are in [bold/italics].

Statistics presented as Mean ± SD, Median [P25, P75], N (column %).

Out of the above listed EEG features in Methods, 3 features were selected to create a feature-set for training binary classification algorithms shown to be significantly different between groups; these included 2 from bandwise coherence generated by channels pairs O2 – C4 for both Delta and Theta bands, and channel O2 for the Beta band in PSD.

Following recent trends in best ML practices^14^, several traditional ML classification algorithms were considered, using cross-validation and a grid-search strategy to find their optimal hyperparameters. The best results were obtained from k-nearest neighbors algorithm (KNN). To ensure data splits were properly distributed between folds, we validated the classifier using a stratified K cross-validation using *k* = 5. Within our dataset with n = 31 for the high ESS group and n = 41 for the low ESS group, we computed the following metrics for validation: accuracy, area under the receiver operating characteristic curve (AUC-ROC), precision, specificity and recall. Accuracy was calculated within the k-folds cross validation by counting the number of out-of-sample predicted labels that matched the true label of the sample, and dividing this total by the number of samples.

**Figure 1:**
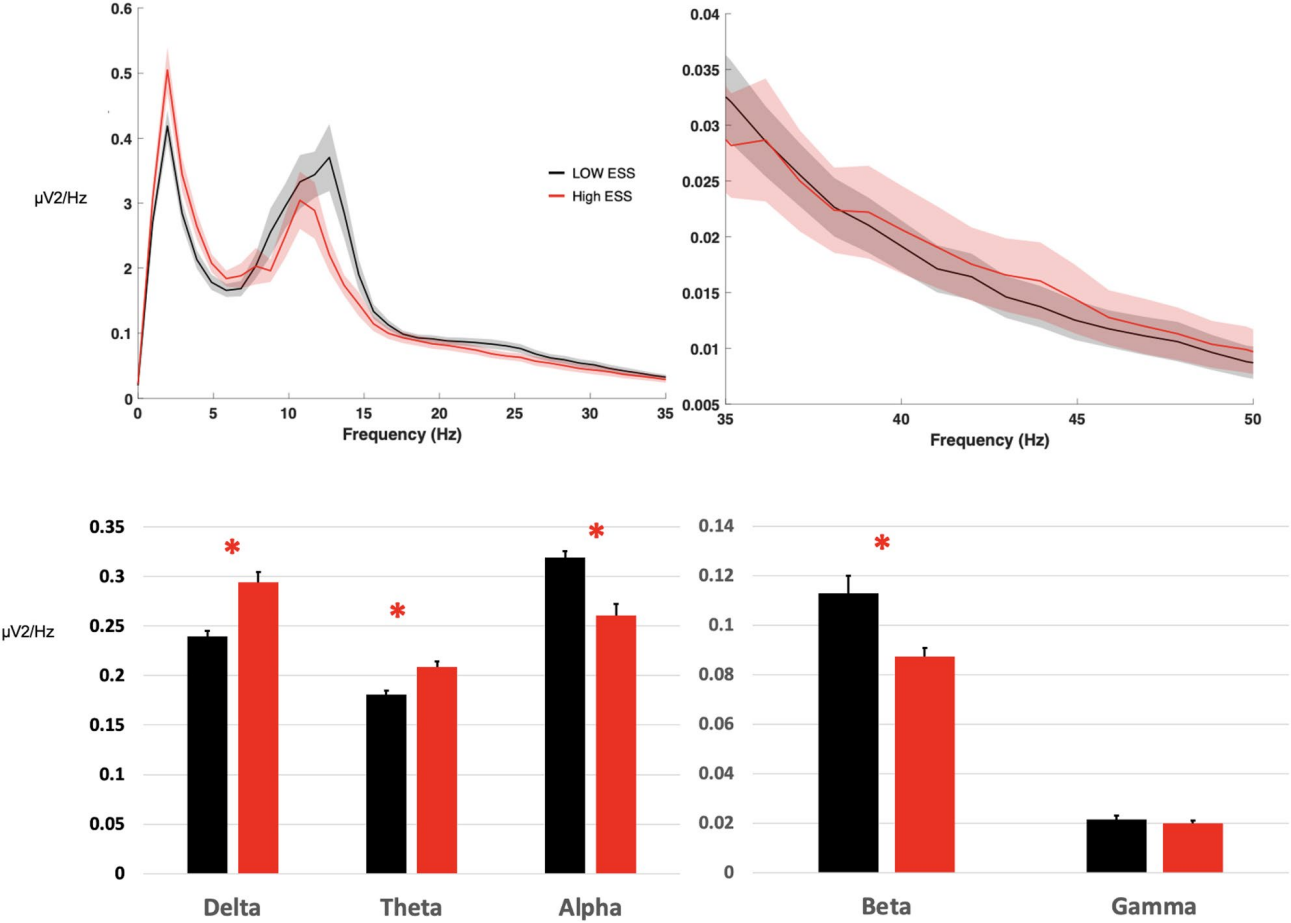
Power Spectral Density (mean of 6 EEG channels) in high ESS (n=31) and low ESS (n=41) subjects in the 0-50 Hz frequency range (upper row). Mean power in the frequency bands delta (1-4 Hz), theta (5-9 Hz), alpha (10-13 Hz), beta (14-32 Hz) and low gamma (33-52 Hz) (lower row).

**Figure 2:**
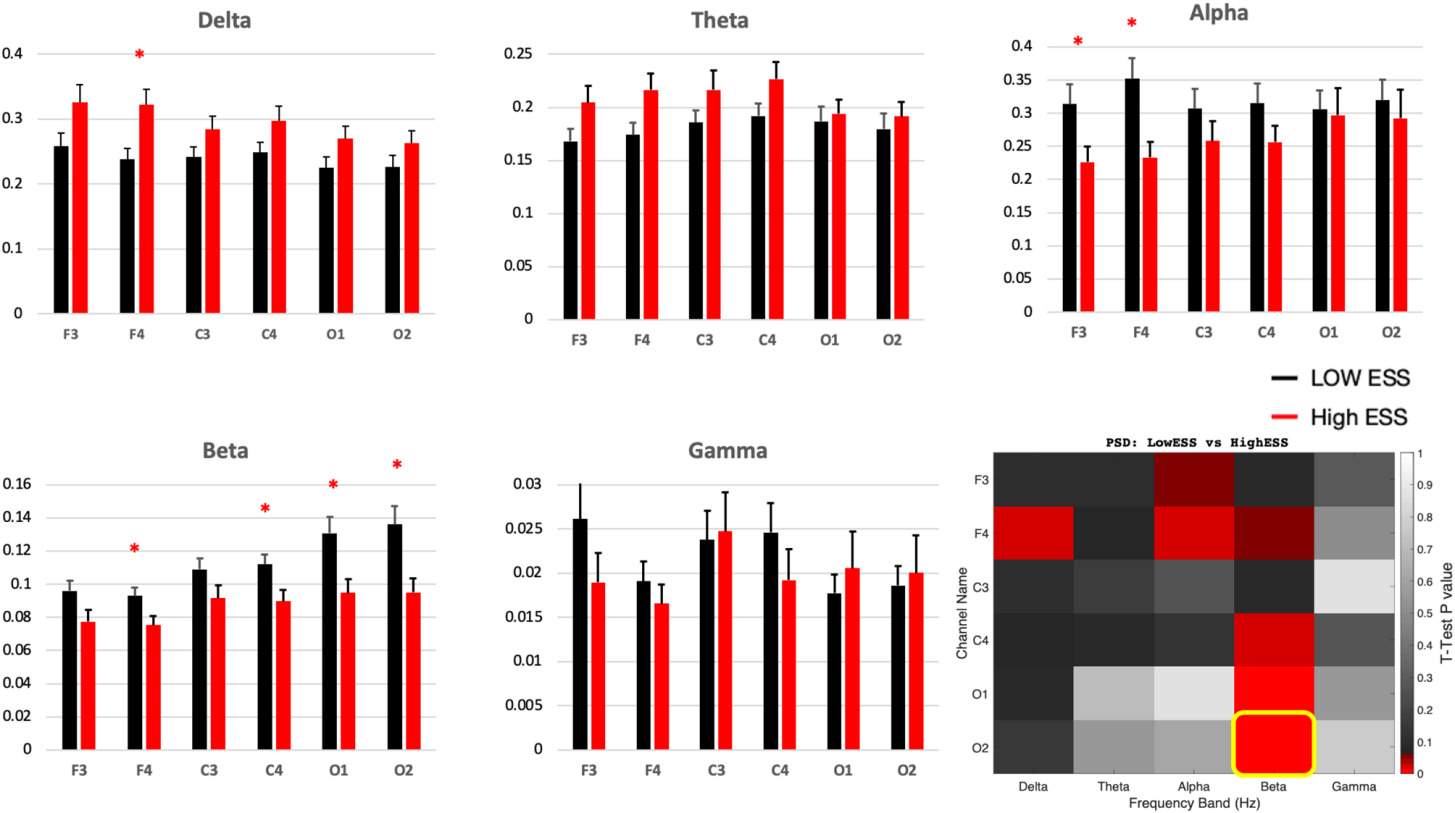
Histograms show Power Spectral Density in 6 individual EEG channels in high ESS (n=31) and low ESS (n=41) subjects in the frequency bands delta (1-4 Hz), theta (5-9 Hz), alpha (10-13 Hz), beta (14-32 Hz) and low gamma (33-52 Hz). Heat map shows corresponding t-test p-values for individual channels in each band (red hue indicates p < 0.05, highlighted cell indicates a feature selected for the training of a ML algorithm).

The spectral density for delta, theta, alpha, and beta power bands showed a statistically significant difference in the mean EEG power in high ESS compared to the low ESS group (Fig. 1), including.an increase in delta (0.294 ± 0.010 in high ESS, 0.239 ± 0.005 in low ESS, *p* < 0.001), in theta (0.208 ± 0.005 in high ESS, 0.181 ± 0.004 in low ESS, *p* < 0.001) and a decrease in alpha (0.260 ± 0.011 in high ESS, 0.318 ± 0.007 in low ESS, *p* < 0.001) and beta (0.087 ± 0.003 in high ESS, 0.112 ± 0.007 in low ESS, *p* < 0.001). Analysis of power in individual 6 channels further showed that significant changes were not localized to particular brain areas. Significant differences in delta, alpha, and beta power bands in each EEG channel were found between low and high ESS groups (Fig. 2).

**Figure 3:**
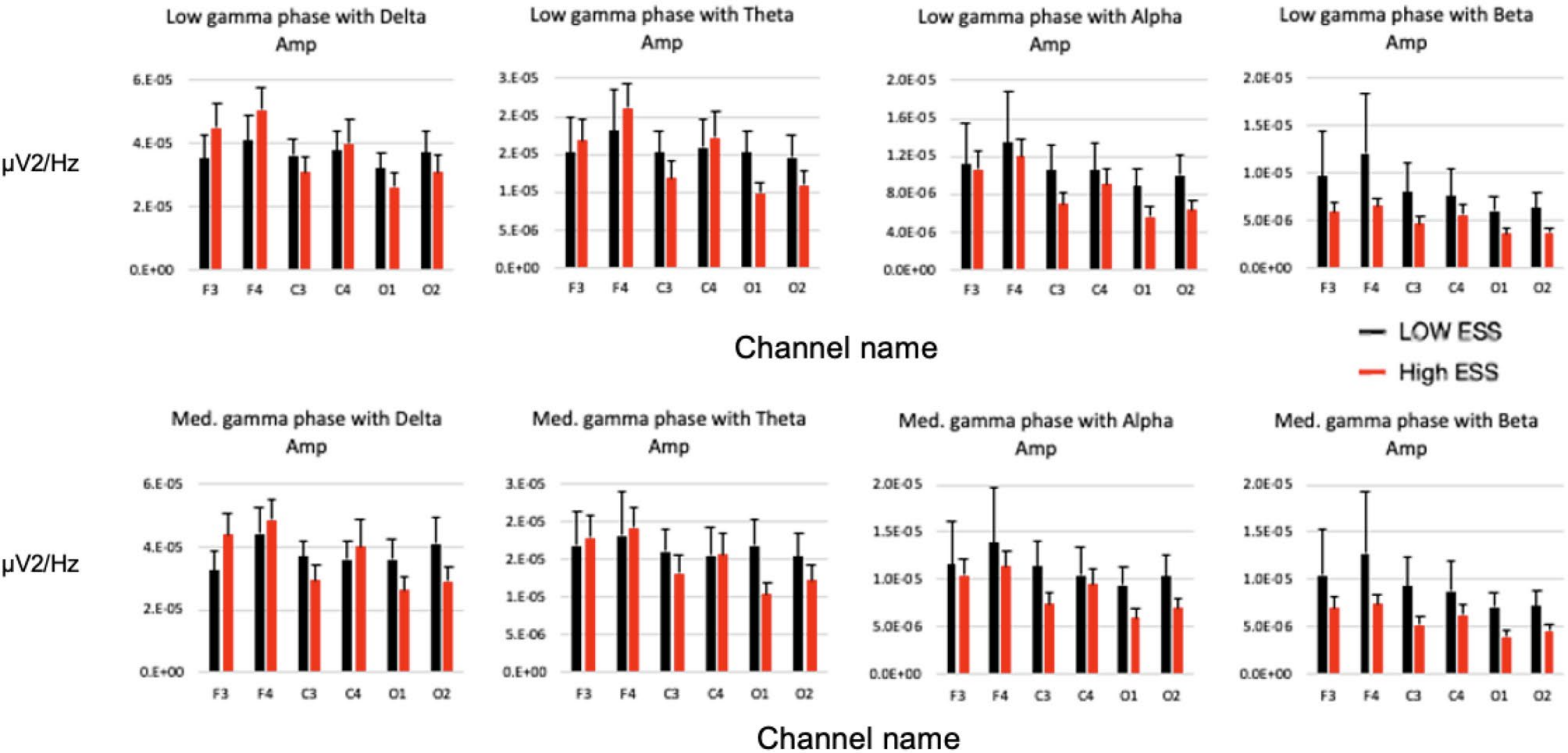
Phase-amplitude coupling (PAC) in 6 individual EEG channels in high ESS (n=31) and low ESS (n=41) subjects between low gamma and delta, theta, alpha, beta respectively (upper row), as well as between medium gamma and delta, theta, alpha, beta respectively (lower row). No statistically significant difference was noted between groups in any individual channel.

For Phase-amplitude coupling (PAC) in 6 individual EEG channels, there was no significant difference between low gamma and delta, theta, alpha, beta respectively, as well as between medium gamma and delta, theta, alpha, beta respectively (Fig. 3).

**Figure 4:**
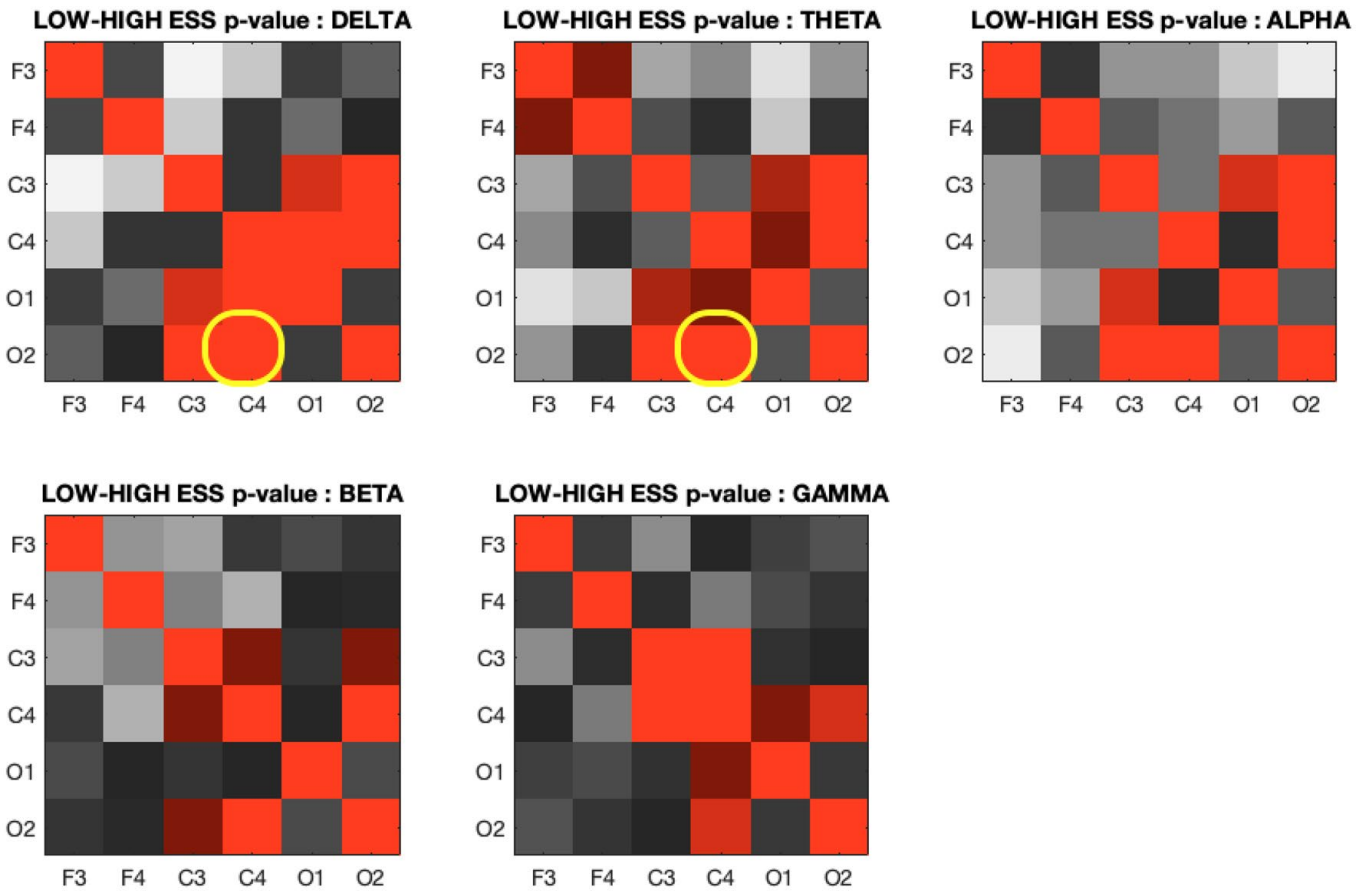
Coherence in 6 individual EEG channels in high ESS (n=31) and low ESS (n=41) subjects. The values are symmetrical across one diagonal (the 2 highlighted cells indicate features that were selected for the training of a ML algorithm).

In Fig. 4, we compared the coherence in 6 individual EEG channels across each frequency band. Significant difference was found for the high ESS and low ESS groups and between coherence in some of the individual channels (C4 and O2).

**Figure 5:**
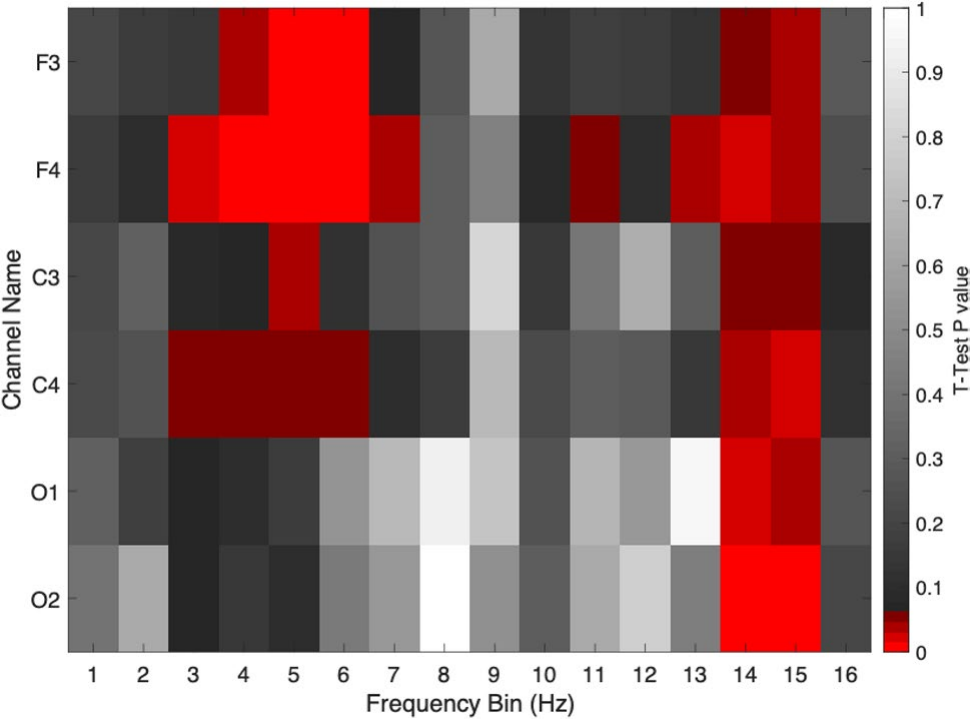
Heat map shows t-test p-values for individual channels, in high ESS (n=31) and low ESS (n=41) subjects, in each frequency bin from 1 Hz to 16 Hz. (red hue indicates p<0.05).

We computed PSD by Hz (Fig. 5), which is similar to Band by Channel (Fig. 2 bottom right), except that instead of bands, we are using bins from 1 to 16 Hz. This yielded to 96 features (16 Hz × 6 channels) to be considered. In Fig. 2 (Heat map (bottom right)), we compared average PSD in 6 individual EEG channels across each frequency bin. Significant difference was found between the high ESS and low ESS groups in channel O2 in Beta band.

The 3 features generated from the PSD and Coherence analysis were used to train k-NN binary classifier of high versus low ESS. Our model reached an accuracy of 80.2%, an AUC-ROC of 79.5%, a precision of 79.2% a recall of 73.8%, and specificity of 85.3%.

**Figure 6:**
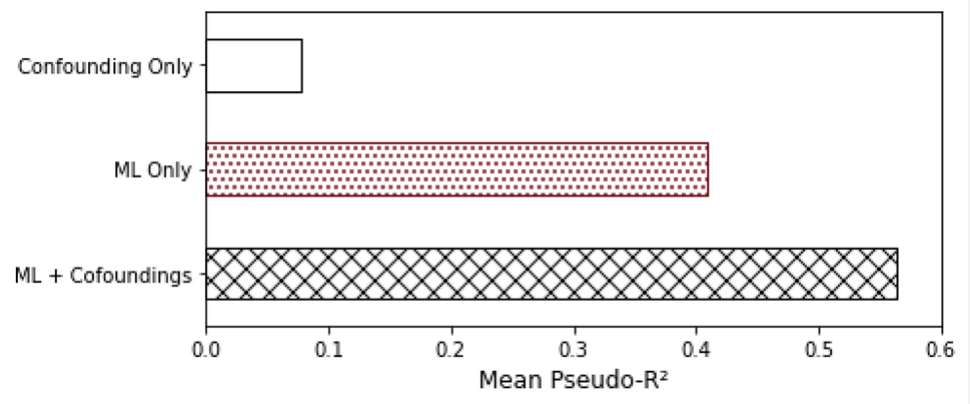
Mean Pseudo R² computed by a generalized linear model on the test sets of cross-validation for 3 scenarios: using only the machine learning prediction, using only the confounding variables age, BMI and gender and using the combination of both machine learning and confounding.

In our analysis of confounding variables that could influence our EEG findings, we compared the predictions of our ML classifier with the potential confounding variables: age, BMI, and gender. After training our model, in each fold of our cross-validation, we used a generalized linear model to predict EDS on the test set in 3 scenarios: Using only our ML output, using only the confounding variables, using both the confounding variables and the ML output. To aggregate the results for the 5 folds, we computed the mean Pseudo-R^2^ and the mean p-values. In Fig. 6, we show the mean Pseudo-R^2^, whereas our ML only reached 41% and the confounding only reached 8%. When combining both ML and confounding variables reached 56%. Table 2 shows the mean *p* values from the generalized linear regression for the same 3 scenarios for each variable. We observed that we have a statistically significant predictor when only the ML output is used (*p* value < 0.05).

**Table 2:** Mean p-values for confounding variables and ML predictions.

	Age	BMI	Gender	ML prediction
Confounding only	0.610	0.516	0.601	–
ML + confounding	0.476	0.612	0.473	0.146
ML only	–	–	–	0.021

## Discussion

Clinically, EDS overlaps with other common sleep and mood disorders that are considered causal to EDS, such as obstructive sleep apnea (OSA), narcolepsy, depression, and post acute sequelae of COVID-19^15^, with an estimated prevalence of 20% of adults in the United States^16^. These causes are usually misdiagnosed or undiagnosed in the population, even when a patient performs a sleep study^7,17^.

Several approaches have been investigated to develop an objective neurophysiologic biomarker capable of capturing symptoms of EDS. For example, the least absolute shrinkage and selection operator (LASSO) was used to predict ESS from EEG signals collected from train drivers, but with varying degrees of success and requiring more complex computational techniques compared to our study, which was guided by a statistical approach for the selection of ML features^18^. Another proposed sleepiness biomarker is the odds ratio product, computed from the delta, theta, alpha-sigma, and beta frequency bands from EEG signals, and its association to ESS^19^. Despite previous work, however, the direct classification of high or low ESS using EEG lacks transparency in the ML approach.

In this study, we demonstrate the feasibility of an automated analytical pipeline, using resting state EEG during wakefulness and ML, of accurately classifying EDS as low versus high. Our results showed that the average power spectrum across EEG channels in low ESS patients is significantly enhanced in the alpha and beta bands and attenuated in the delta and theta bands compared to high ESS subjects.

After analyzing sleep EEG in two groups of patients, we identified significant differences in the delta, theta, alpha, and beta frequency bands between those who reported high versus low sleepiness. Aiming to estimate daytime sleepiness at the individual level, we trained a k-NN classifier reaching 80.2% accuracy, 79.2% precision, 73.8% recall, and 85.3% specificity. Thus, our work demonstrates a potential for EEG analysis to generate biomarkers for excessive daytime sleepiness and the use of signal processing and ML to classify sleepiness at the individual level. Moreover, we emphasized the novelty of these biomarkers when we showed that even after controlling for confounding variables, ML predictors alone significantly explain EDS.

These results suggest that short duration, resting state, wake EEG contains information that could be leveraged to reliably assess EDS, thereby enhancing clinical care. These findings should be contextualized with the historical standard of utilizing EEG patterns to qualitatively and to a lesser extent quantitatively define sleep stages as per the American Academy of Sleep Medicine and assessment of symptoms of excessive daytime sleepiness with time-intensive objective tests such as the multiple sleep latency test^20^.

Our results show that low ESS subjects have higher EEG activity in the beta band in the occipital region than high ESS subjects. Previous research has also highlighted the significant differences between these biomarkers in groups of EDS patients^21^. Others have suggested that neural mechanisms related to visual tasks and attention may be linked to these biomarkers. For instance, an increase in response time for visual tasks among students in a real classroom is associated with a decrease in the mean baseline beta band power over the occipital region^22^. Also, in^23^, beta band activity in the occipital region was linked to attention deficits in elderly subjects, with increased activity preceding correct responses for visual tasks.

Some limitations of our work deserve attention. Since the sample size could be considered relatively small (n = 72) in the field of EEG/ML^14^, our model would benefit from validation against a prospective dataset as per ML best practices, as well as across different geographical sites. Our approach still depends on manual sleep staging annotation since the automated pipeline does not determine "wake" epochs. Still, the “wake” period is determined after we scan the data looking for the manual annotations of "lights out," "wake," and sleep stage "N1". Moreover, according to AASM, the awake epochs can range from full alertness through early stage of drowsiness. We also used signal processing techniques to extract EEG features, for example power in predefined delta, theta, alpha, beta, and gamma bands. This may have limited our feature space for biomarkers in contrast to other techniques that are based on training deep learning models directly from EEG raw signals^24^, although such techniques are non-transparent and more computationally demanding. We also acknowledge that some demographic variables might have an impact on EEG, such as age^25–28^ and gender^29,30^, and we did not specifically control for such parameters, although mean demographic values were overall comparable.

We chose extremes of subjective perception of dozing propensity defined by ESS as an exploratory use case for algorithm development. The sleep studies were not selected based upon obstructive sleep apnea status, but rather patients were most often referred for the sleep study based upon suspected OSA with varying levels of self-reported sleepiness. Therefore, there are opportunities to build upon this work in the future by examining EEG-based biomarkers which reflect the spectrum of sleepiness.

Finally, we envision that our automated and quantitative method for assessing EDS can be operationalized by adding our method's output into patients' Electronic Health Record after performing a polysomnogram test. Future investigation should focus on whether a point of care measure such as this could be used to risk stratify patients to identify those for example who may need drowsy driving education or to identify sleep phenotypes more responsive to specific interventions such as pharmacotherapeutics to treat hypersomnia disorders.

## Conclusion

In this study, we investigated the potential of using EEG signals recorded during the awake period in polysomnograms as biomarkers for excessive daytime sleepiness. We identified significant differences in the delta, theta, alpha, and beta frequency bands between those who reported high versus low sleepiness measured by the widely used ESS. Aiming to estimate daytime sleepiness at the individual level, we trained a k-NN ML classifier that reached 80.2% accuracy, 79.2% precision, 73.8% recall, and 85.3% specificity in a retrospective cohort. After controlling for potential confounding variables, we show that this study innovates in building a direct association between EEG and ESS. Ultimately, we provide powerful techniques for those areas that can leverage advanced EEG analysis, such as underdiagnosed EDS studies.

## Data Availability

The data that support the findings of this study and the datasets generated and analyzed are not publicly available due to human subject concerns, but are available from the corresponding author on reasonable request.
