# Author Correction: Machine learning polysomnographically-derived electroencephalography biomarkers predictive of epworth sleepiness scale

**DOI:** 10.1038/s41598-023-37716-7

**Published:** 2023-06-30

**Authors:** Matheus Araujo, Samer Ghosn, Lu Wang, Nengah Hariadi, Samantha Wells, Carl Y. Saab, Reena Mehra

**Affiliations:** 1 Sleep Disorders Center, Neurological Institute, Cleveland Clinic Foundation, Cleveland, OH USA; 2 Department of Biomedical Engineering, Cleveland Clinic Foundation, Cleveland, OH USA; 3 Quantitative Health Sciences, Lerner Research Institute, Cleveland Clinic Foundation, Cleveland, OH USA; 4 Metro Health Medical Center, Cleveland, OH USA; 5 Department of Biomedical Engineering, Brown University, Providence, RI USA; 6 Respiratory Institute, Cleveland Clinic Foundation, Cleveland, OH USA; 7 Cardiovascular and Metabolic Sciences, Lerner Research Institute, Cleveland Clinic Foundation, Cleveland, OH USA; 8 Heart and Vascular Institute, Cleveland Clinic Foundation, Cleveland, OH USA

Correction to: *Scientific Reports* 10.1038/s41598-023-34716-5, published online 05 June 2023

The original version of this Article contained errors in the names of the authors Matheus Araujo, Samer Ghosn, Lu Wang, Nengah Hariadi, Samantha Wells, Carl Y. Saab & Reena Mehra, which were incorrectly given as Araujo Matheus, Ghosn Samer, Wang Lu, Hariadi Nengah, Wells Samantha, Saab Y. Carl & Mehra Reena.

The original Article has been corrected.

